# Phylogenetic and Drug-Resistance Analysis of HIV-1 Sequences From an Extensive Paediatric HIV-1 Outbreak in Larkana, Pakistan

**DOI:** 10.3389/fmicb.2021.658186

**Published:** 2021-08-17

**Authors:** Syed Hani Abidi, George Makau Nduva, Dilsha Siddiqui, Wardah Rafaqat, Syed Faisal Mahmood, Amna Rehana Siddiqui, Apsara Ali Nathwani, Aneeta Hotwani, Sharaf Ali Shah, Sikander Memon, Saqib Ali Sheikh, Palwasha Khan, Joakim Esbjörnsson, Rashida Abbas Ferrand, Fatima Mir

**Affiliations:** ^1^Department of Biological and Biomedical Sciences, Aga Khan University, Karachi, Pakistan; ^2^Department of Translational Medicine, Lund University, Lund, Sweden; ^3^Kenya Medical Research Institute-Wellcome Trust Research Programme, Kilifi, Kenya; ^4^Medical College, Aga Khan University, Karachi, Pakistan; ^5^Department of Medicine, Aga Khan University, Karachi, Pakistan; ^6^Department of Community Health Sciences, Aga Khan University, Karachi, Pakistan; ^7^Department of Pediatrics and Child Health, Aga Khan University, Karachi, Pakistan; ^8^Bridge Consultants Foundation, Karachi, Pakistan; ^9^Sindh AIDS Control Program, Ministry of Health, Karachi, Pakistan; ^10^Department of Clinical Research, London School of Hygiene & Tropical Medicine, London, United Kingdom; ^11^The Nuffield Department of Medicine, University of Oxford, Oxford, United Kingdom

**Keywords:** HIV-1, outbreak investigation, phylogenetic analysis, drug resistance, paediatric [MeSH]

## Abstract

**Introduction:**

In April 2019, an HIV-1 outbreak among children occurred in Larkana, Pakistan, affecting more than a thousand children. It was assumed that the outbreak originated from a single source, namely a doctor at a private health facility. In this study, we performed subtype distribution, phylogenetic and drug-resistance analysis of HIV-1 sequences from 2019 outbreak in Larkana, Pakistan.

**Methods:**

A total of 401 blood samples were collected between April–June 2019, from children infected with HIV-1 aged 0–15 years recruited into a case-control study to investigate the risk factors for HIV-1 transmission. Partial HIV-1 *pol* sequences were generated from 344 blood plasma samples to determine HIV-1 subtype and drug resistance mutations (DRM). Maximum-likelihood phylogenetics based on outbreak and reference sequences was used to identify transmission clusters and assess the relationship between outbreak and key population sequences between and within the determined clusters. Bayesian analysis was employed to identify the time to the most recent common recent ancestor (tMRCA) of the main Pakistani clusters.

**Results:**

The HIV-1 circulating recombinant form (CRF) 02_AG and subtype A1 were most common among the outbreak sequences. Of the treatment-naïve participants, the two most common mutations were RT: E138A (8%) and RT: K219Q (8%). Four supported clusters within the outbreak were identified, and the median tMRCAs of the Larkana outbreak sequences were estimated to 2016 for both the CRF02_AG and the subtype A1 clusters. Furthermore, outbreak sequences exhibited no phylogenetic mixing with sequences from other high-risk groups of Pakistan.

**Conclusion:**

The presence of multiple clusters indicated a multi-source outbreak, rather than a single source outbreak from a single health practitioner as previously suggested. The multiple introductions were likely a consequence of ongoing transmission within the high-risk groups of Larkana, and it is possible that the so-called Larkana strain was introduced into the general population through poor infection prevention control practices in healthcare settings. The study highlights the need to scale up HIV-1 prevention programmes among key population groups and improving infection prevention control in Pakistan.

## Introduction

The HIV-1 pandemic has been established for 40 years and has resulted in approximately 32.7 million deaths worldwide (de Mendoza, 2019). One of the characteristic features of HIV-1 is its high mutation rate and recombination rate within and between hosts, leading to the emergence of distinct subtypes and circulating recombinant forms (CRFs) (Taylor et al., 2008; van Zyl et al., 2018). The subtypes can also recombine to give rise to unique recombinant forms (URFs) and minor HIV-1 variants (Taylor et al., 2008). The consequent genetic diversity that characterises HIV-1 infection has implications for virological control and transmission. Mutations encoded by the virus can interfere with epitope processing and recognition, leading to immune evasion. In addition, mutations may also lead to resistance to anti-retroviral drugs (van Zyl et al., 2018). Furthermore, certain immune- or drug-escape mutations may facilitate rapid transmission (van Zyl et al., 2018).

The phylogenetic and phylodynamic analysis of sequences derived from people living with HIV-1 (PLWH), particularly those who are part of an outbreak, can help answer fundamental questions such as the directionality and pattern of transmission and in understanding the introduction of HIV-1 into different regions, as well as identify clusters of transmission (Kosakovsky Pond et al., 2018; Dawson et al., 2020; Romero-Severson et al., 2020).

Pakistan has experienced a growing HIV-1 epidemic that is concentrated among three key population groups namely persons who inject drugs (PWID), transgender sex workers [also known as *Hijra* sex workers (HSW)], and men who have sex with men (MSM) (Raees et al., 2013) in whom prevalence is around 38–40, 11, and 7.5%, respectively (Baqi et al., 1998; Waheed and Waheed, 2017; Hasan et al., 2018). Only 36,000 of an estimated 160,000 PLWH in Pakistan were aware of their HIV-1 positive status in 2020, and only 24,606 PLWH were receiving HIV-1 treatment of whom 7,693 were PWID (Esbjörnsson et al., 2016).

In April 2019, a cluster of fourteen HIV-1 diagnoses in children was reported in Ratodero, a town in Larkana district, Pakistan (Siddiqui et al., 2020). By December 2019, 1,167 children have been diagnosed with HIV-1 through a screening programme established in response to the outbreak (Brenner et al., 2007). Larkana has had three previous outbreaks of HIV, the first among PWID in 2003, the second in 2016 among 12 children in a paediatric hospital, and the third in 2016 among 56 individuals in a renal dialysis unit (Brenner et al., 2007; Altaf et al., 2016). These outbreaks were linked to poor infection prevention control practices including reuse of needles and inadequate blood screening.

For Pakistan, the outbreak in 2019 is unprecedented in terms of predominantly affecting children and its magnitude: prior to the outbreak, 1041 children had ever registered for HIV-1 care nationally over the past 13 years (Mir et al., 2020a). Early during the outbreak, media reports implicated a local doctor who had treated several of the infected children and who was later diagnosed with HIV, in spreading HIV-1 infection (Abi-Habib and Masood, 2019; Siddiqui et al., 2020).

In this study, we conducted a phylogenetic analysis to investigate the HIV-1 outbreak subtype and the pattern and source of transmission, and specifically whether this was a single-source outbreak. Furthermore, we also analysed the sequences for presence of drug resistance mutations (DRMs).

## Materials and Methods

### Study Design and Setting

This study was embedded in an individually matched case-control study that recruited 401 cases defined as children aged 0–15 years who registered for HIV-1 care at the Paediatric Treatment Center at Shaikh Zayed Children’s Hospital (Siddiqui et al., 2020). This centre was established by the Sindh AIDS Control Program in response to the outbreak. Prior to the outbreak, the nearest paediatric HIV-1 services were in the provincial capital, Karachi, situated more than 400 kilometres from Ratodero. Age-, sex-, and neighbourhood-matched HIV-uninfected controls were also recruited. An interviewer-administered questionnaire collected data on risk factors for HIV-1 infection. A blood sample was collected for Hepatitis B and C serology, and for HIV-1 phylogenetic studies (in cases only). Written informed consent was obtained from guardians and assent from participants. The study was approved by the Aga Khan University Ethical Review Committee (ERC# 2019-1536-4200). Prior to sample collection, written informed consent was obtained from the guardians, and if the child was able to understand the study procedures, a written assent was obtained. The study objectives were explained to the patient at the time of taking consent/assent and patients were informed that their identities will remain confidential. The participants were also informed that they had the opportunity to withdraw from the study at any given time and that this would have no consequences on the treatment or the care that they would receive.

### DNA Amplification and HIV-1 Genotyping

Proviral DNA was extracted from blood samples obtained from cases using Qiagen’s QIAamp DNA blood mini kit according to the manufacturer’s instructions and stored at -80°C. The *pol* gene was amplified from each extracted DNA sample using a two-step nested polymerase chain reaction (PCR) strategy. Two sets of outer primers were used: Forward (POLOF CAGCATGYCAGGGAGTRGGRGGACC, amino acid; 1832-1856, HXB2, IBF1 5′-AAATGATGACAGCATGTCAG GGAGT-3′. nt 1823-1847, HXB2) and Reverse (IBR1 5′-AACTT CTGTATATCATTGACAGTCCA-3′. nt 3303-3328, HXB2). The first-round product was used as a template for the second round with primer set, Forward (POLIF 5′-AGGCTAATTTT TTAGGGAARATYTGGCCTTCC-3′. nt 2078-2109, amino acid PR: 1–9; HXB2) and reverse (RTOUT3 5′-TATGTCATT GACAGTCCAGCT-3′. nt 3300–3320, amino acid RT: 251–257 HXB2) (Tariq et al., 2018). PCR Mastermix (ABM) Bestaq (2X) cat# G464 and Hotstart (2X) cat# G906 were used to prepare a 25 ul reaction mixture, and 0.8 pmol and 0.6 pmol primer used for the first and second round, respectively. Thermo cycle conditions were as follows: denaturation at 95°C for 5 min, followed by 40 cycles of denaturation at 95°C for 1 min, annealing at 50°C for IBF1/IBR1 and 55°C for POLOF/IBR1 sets (round 1), 60°C (round 2) for 20 s, extension at 72°C for 1 min with a final extension of at 72°C for 7 min. The protocol was run with positive and negative control to confirm results. The amplicons underwent sequencing using the Sanger sequencing platform (Macrogen, South Korea) and the sequences were deposited in the GenBank and assigned the accession numbers MN698251-MN698253, MN698255-MN698264, MN752136, MN752137, and MT748850-MT749178.

### Subtype Analysis

HIV-1 *pol* sequence data used in the study comprised of either newly generated sequences (referred to as outbreak sequences) or Pakistani HIV-1 *pol* sequences retrieved from the Los Alamos HIV sequence database^1^ (referred to as published Pakistani sequences representing Pakistani people who inject drugs (PWID), heterosexuals, sex workers, and other individuals with unknown transmission risk) (Kuiken et al., 2003). Outbreak sequences and published Pakistani HIV-1 *pol* sequences (referred to as the Pakistani dataset) were aligned with HIV-1 Group M (subtypes A-K + Recombinants) subtype reference sequences^1^ using the MAFFT algorithm in Geneious Prime 2019 (Larkin et al., 2007). Subtyping of each sequence was determined by maximum-likelihood (ML) phylogenetic analysis in PhyML using the general time-reversible substitution model with a gamma-distributed rate variation and proportion of invariant sites (GTR + Γ4 + I) (Guindon et al., 2010). Branch support was estimated using the approximate likelihood ratio test with the Shimodaira-Hasegawa-like procedure (SH-aLRT) in PhyML, where SH-aLRT support values ≥0.90 were considered significant (Guindon et al., 2010). Phylogenies were visualised in FigTree v1.4.4.^2^

### Cluster Analysis

To identify local transmission clusters, subtype-specific ML phylogenies were reconstructed for the main/predominant HIV-1 strains identified in the outbreak. The most similar non-Pakistani sequences for each sequence in the Pakistani dataset were retrieved from the NCBI GenBank and used as reference sequences as previously described (Esbjörnsson et al., 2016; Nazziwa et al., 2020; Nduva et al., 2020). The Pakistani dataset and GenBank reference sequences were aligned by subtype or CRF and subtype/CRF-specific phylogenies were reconstructed in PhyML (Guindon et al., 2010). Monophyletic clusters having aLRT-SH ≥0.90 and comprising ≥80% Pakistani sequences were defined as Pakistani-specific clusters (Esbjörnsson et al., 2016; Hassan et al., 2017; Nazziwa et al., 2020; Nduva et al., 2020). Clusters were classified into dyads (2 sequences), networks (3–14 sequences), or large clusters (>14 sequences) (Esbjörnsson et al., 2016).

### Estimating Dates of the Most Recent Common Ancestor for Each Cluster

The dates of origin (time to the most recent common ancestor; tMRCA) of the large Pakistani-specific HIV-1 clusters were estimated using Bayesian Markov Chain Monte Carlo (MCMC) inference in BEAST (v1.10.4) (Gill et al., 2020). All Larkana outbreak sequences were sampled in 2019 and did not independently have a sufficient temporal signal for inference of the dates of origin. Hence, supplementary non-Pakistani reference sequences (seven CRF02_AG and six sub-subtype A1 sequences), and Pakistani sequences from previous outbreaks (33 CRF02_AG and 11 sub-subtype A1 sequences) sampled from different years were used to inform the temporal signal (assessed in TempEst v1.5.3) (Rambaut et al., 2016). Subtype-specific Bayesian inferences were done in BEAST 1.10.4 using the Bayesian Skygrid model with an uncorrelated lognormal relaxed clock and inferred under the GTR + Γ4 + I substitution model (Drummond et al., 2005; Baele et al., 2012; Gill et al., 2013; Suchard et al., 2018). BEAST runs of 500 million generations were performed, sampling every 50,000th iteration, and discarding the first 10% of samples as burn-in. Convergence was determined in Tracer v.1.7.0, defined as effective sample sizes (ESS) ≥200 (Suchard et al., 2018). Maximum clade credibility (MCC) trees were summarised in Tree-Annotator v1.10.4 and visualised in Figtree (v1.4.4).

### Drug Resistance Mutation Analysis

Mutations in the HIV-1 *pol* gene (protease and reverse transcriptase region) associated with resistance against protease and reverse transcriptase inhibitors was determined using the Stanford HIV-1 drug resistance database (Rhee et al., 2003), and confirmed using the 2019 Update of the Drug Resistance Mutations in HIV-1 by the International AIDS Society–United States (Wensing et al., 2019). The DRMs were also classified as those conferring high, intermediate, low, and potential low-level resistance using the algorithm described in Stanford HIV-1 drug resistance database and IAS-United States report.

## Results

### Study Population

Of the blood samples obtained from the 401 cases enrolled in the case-control study, 344 were successfully amplified and sequenced. The remaining 57 samples failed to amplify possibly due to low viral load secondary to receiving antiretroviral treatment or due to genomic diversity attributed to quasi-species in an individual (Debyser et al., 1998; Gupta et al., 2017). Out of 344 cases, socio-demographic information was available for 321 sequences, while information for 23 cases was missing. The median age of participants was three (IQR: 2–5) years and 65% were male (Table 1). The majority (84.4%) were taking ART at the time of sampling for a median period of 41 days (range: 22–192 days). The ART regimen comprised of zidovudine, lamivudine, and nevirapine. Most participants lived in Ratodero, the epicentre of the outbreak, while the remainder were from other areas of Larkana district and neighbouring districts (Table 1).

**TABLE 1:** Characteristics of study participants.

**Category/variable**	**Total no. (%)**
**Age (years)**	
0–5	248 (77%)
5–10	58 (18%)
10–15	15 (5%)
**Sex**	
Male	208 (65%)
Female	113 (35%)
**Location**	
Ratodero, Larkana	132 (41%)
Outside Ratodero but within Larkana district	141 (44%)
Shikarpur district	44 (13.7%)
Jafarabad district	1 (0.3%)
Khairpur district	1 (0.3%)
Nawabshah district	1 (0.3%)
**ART History**	
Naïve	50 (15.6%)
Experienced	271 (84.4%)
**ART duration** (268)	
<30 days	93 (28.97%)
>30–180 days	175 (54.5%)
**HCVand HBV co-infections**	
HBV positive	75 (23.4%)
HCV positive	26 (8%)
**Maternal HIV-1 status**	
HIV-1 positive mother	28 (8.7%)
Mother’s HIV-1 status unknown	4 (1.2%)

### Pakistani HIV-1 Sequence Dataset and HIV-1 Subtypes and CRFs

**FIGURE 1:**
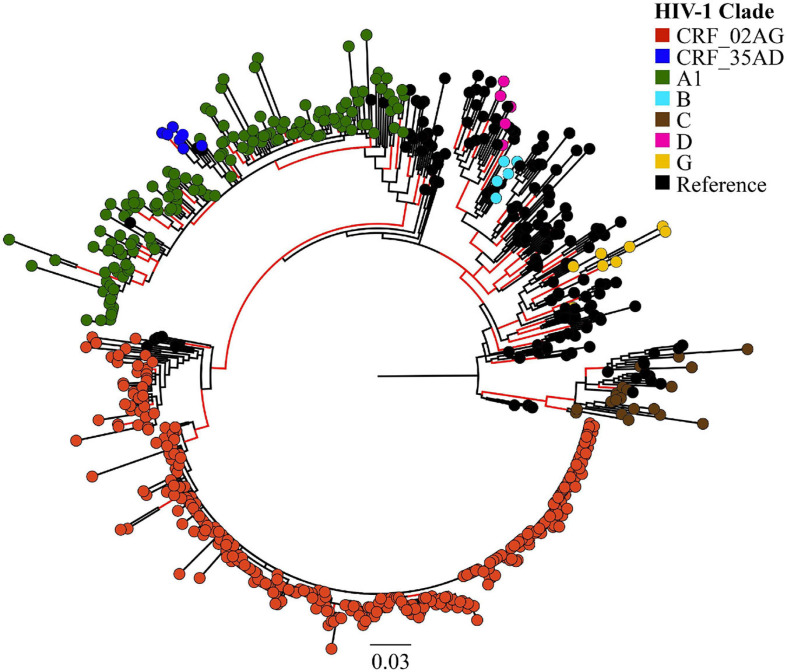
Maximum likelihood (ML)-based subtyping of HIV-1 sequences: ML-based phylogeny/subtyping of 532 HIV-1 sequences (including 344 outbreak sequences from Larkana-Pakistan). Branch tips on the phylogenetic tree are coloured according to HIV CRF/subtype (Red: CRF_02AG; Blue: CRF_35AD, Green: subtype A1; Sky Blue: subtype B; Brown: subtype C; Pink: subtype D; Yellow: subtype G; Black: reference sequences from the Los Alamos HIV database). Scale bar units are nucleotide substitutions per site.

Overall, we analysed 532 HIV-1 partial *pol* sequences in the Pakistani dataset including outbreak sequences (*N* = 344) and previously published sequences (*N* = 188). HIV-1 CRF02_AG (*N* = 338, 63.5%) dominated the outbreak, followed by sub-subtype A1 (*N* = 149, 28.0%). Additional subtypes found in the outbreak were subtype C (*N* = 20, 3.8%), subtype G (*N* = 8, 1.5%), CRF35_AD (*N* = 7, 1.3%), subtype B (*N* = 5, 0.9%), and subtype D (*N* = 5, 0.9%, Figure 1).

## Identification of Pakistani-Specific HIV-1 Transmission Clusters

**FIGURE 2:**
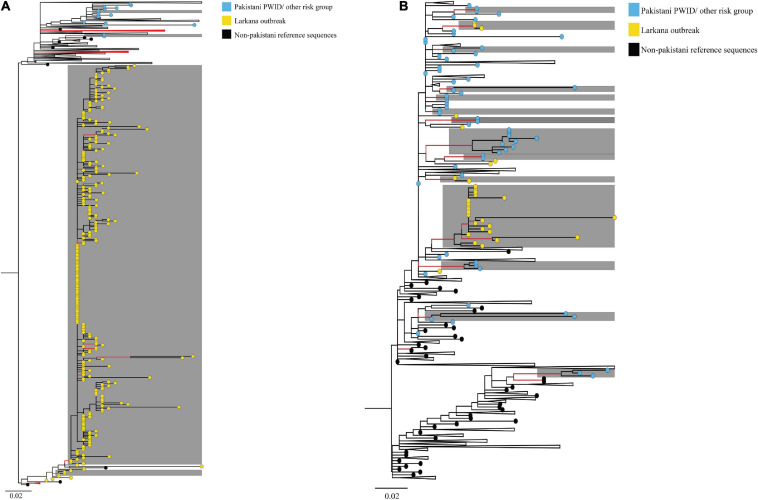
Maximum-likelihood trees summarising clustering patterns of different risk groups in Pakistani: Trees represent **(A)** CRF02_AG, and **(B)** sub-subtype A1 transmission clusters, respectively. Each phylogeny is rooted at the midpoint, and branches are arranged in increasing node order. Branches with aLRT-SH support ≥0.9 are coloured red. Monophyletic clusters with aLRT-SH support ≥0.9 and which have ≥80% sequences from Pakistan are highlighted in grey. To enhance cluster visualisation, some branches containing either reference sequences or Pakistani sequences that are not part of clusters have been collapsed (shown as black triangles, with the recent end of the triangle indicating the latest sampling date). Branch tips within respective clusters are coloured as per cluster risk group (Bluish- green: MSM; Sky blue: Pakistani PWID/other risk groups IDU; Yellow: Larkana paediatric sequences; and Black: Non-Pakistani Reference sequences). Scale bars represent the genetic distance in substitutions per site in both phylogenies. As an overview, among CRF02_AG sequences, whereas PWID and other risk groups formed small clusters (size range 2–4 sequences per cluster), paediatric sequences from the Larkana outbreak formed one large cluster (*N* = 283 sequences). Likewise, among subtype A1 sequences, PWID and individuals from other risk groups formed several small clusters (size range, 2–9 sequences per cluster), whilst paediatric sequences from the Larkana outbreak formed one large cluster (*N* = 22 sequences).

ML phylogenies were reconstructed independently for CRF02_AG (*N* = 338) and sub-subtypes A1 (*N* = 149), which were the most prevalent HIV-1 strains in the outbreak. The final reference dataset comprised of 310 non-Pakistani reference sequences for CRF02_AG, and 382 for sub-subtype A1 remained. Overall, 291 (86.1% CRF02_AG outbreak sequences) and 59 (40.0% sub-subtype A1 outbreak sequences) sequences formed 17 supported Pakistani clusters (size range: 2–283 sequences per cluster). These 17 clusters included 9 dyads (52.9% of all clusters), six networks (33.3%), and two large clusters (11.8%, Table 2 and Figures 2A,B). Sub-subtype A1 clusters were more common (*N* = 13, 76.5%) as compared to the CRF02_AG clusters (*N* = 4, 23.5%). Of the 344 outbreak sequences, 312 (90.7%) were found in four distinct clusters. More specifically, 283 sequences were found in one large CRF02_AG cluster (Figure 2A), two sequences in a CRF02_AG dyad (Figure 2A), 22 sequences in one large sub-subtype A1 cluster (Figure 2B), and four sequences in a sub-subtype A1 network (Figure 2B). HIV-1 clusters of outbreak sequences showed no evidence of mixing with other HIV-1 risk groups of Pakistan. Instead, the Pakistani sequences that were not part of the outbreak formed an exclusive Pakistani cluster of non-outbreak sequences (Table 2).

**TABLE 2:** The number of Pakistani transmission clusters by cluster size, risk group, and shared drug resistance mutations.

**Cluster name**	**HIV-1 subtype**	**Number of Tips**	**Transmission group**	**Number with shared DRM**
Cluster_A1_1	A1	22	Paediatric	RT:E138A; *N* = 21
Cluster_A1_2	A1	3	Paediatric	RT:E138A; *N* = 3
Cluster_A1_3	A1	2	Unknown	
Cluster_A1_4	A1	2	MSM/unknown	
Cluster_A1_6	A1	2	PWID	
Cluster_A1_7	A1	2	Unknown	
Cluster_A1_8	A1	2	Unknown	
Cluster_A1_9	A1	2	Unknown	
Cluster_A1_10	A1	9	PWID/unknown	
Cluster_A1_11	A1	2	Unknown	
Cluster_A1_12	A1	5	Unknown	
Cluster_A1_15	A1	3	MSM/unknown	
Cluster_A1_16	A1	3	CWSW/unknown	
Cluster_CRF02AG_1	CRF02_AG	283	Paediatric	RT:K219Q; *N* = 5, RT:K103N; *N* = 19, RT:E138A; *N* = 7, RT:V17; *N* = 10
Cluster_CRF02AG_2	CRF02_AG	4	Paediatric	RT:E138A; *N* = 1, RT:V17; *N* = 2
Cluster_CRF02AG_3	CRF02_AG	2	Unknown	
Cluster_CRF02AG_4	CRF02_AG	2	Unknown	

*Abbreviations: MSM, men who have sex with men; PWID, people who inject drugs; CWSW, female sex worker; Unknown, sequences lacking information on risk group or transmission route; Outbreak, sequences collected from the 2019 Larkana outbreak; RT, HIV-1 reverse transcriptase gene.*

### Estimation of Time to the Most Recent Common Ancestor (tMRCA)

**FIGURE 3:**
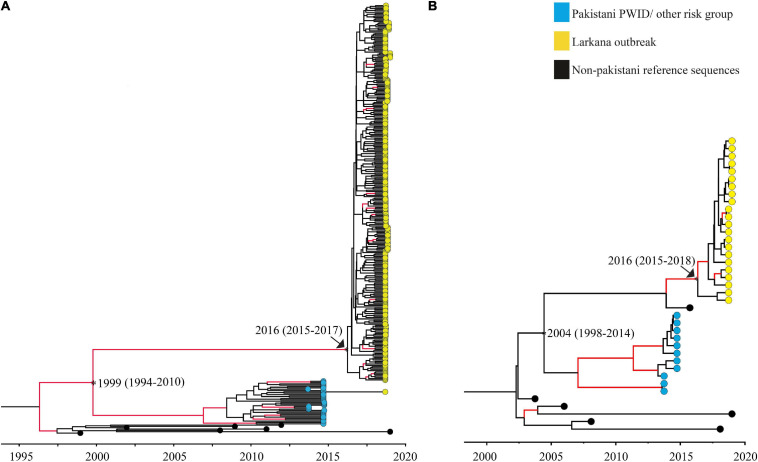
Maximum clade credibility trees used to date clusters: Maximum clade credibility (MCC) trees used to determine the time to the most recent common ancestor of the Pakistani clusters. Trees represent **(A)** the large CRF02_AG outbreak cluster, and **(B)** the large sub-subtype A1 outbreak cluster, respectively. Nodes representing divergence (tMRCA; median and 95% HPD estimates) between the Larkana outbreak and prevailing epidemic sequences (representing PWID and other risk groups) are highlighted as asterisks only. Nodes representing the tMRCA of all outbreak sequences per sub-type/CRF have been highlighted using both an arrow and an asterisk. Branches with posterior value ≥0.9 are coloured red.

The tMRCAs were estimated for the two large clusters (one CRF02_AG and one subtype A1 cluster) comprising of the outbreak sequences. The median tMRCA of the CRF02_AG cluster was estimated to 2016 (95% higher posterior density [HPD] interval: 2015–2017), and the tMRCA of the sub-subtype A1 cluster was estimated to be 2016 (95% HPD interval: 2015–2018). In addition, the divergence time between the outbreak sequences and that of other high-risk groups in Pakistan, such as PWID, was dated to the year 1999 (95% HPD interval: 1994–2010) for the CRF02_AG cluster, and 2004 (95% HPD interval: 1998–2014) for sub-subtype A1 cluster (Figure 3).

### Drug Resistance Mutation Analysis

Drug resistance mutation analysis was conducted on both ART-naïve (15.6%) and ART- experienced (84.4%) sequences from the outbreak. Among ART naïve individuals, 15 (30%) had drug resistance mutations (DRM); the most common of which were the Reverse Transcriptase (RT):E138A (8.0%) and RT:K219Q (8.0%) mutations. Among treatment-experienced individuals, the most common mutations were RT:E138A (12.92%), RT:K219Q (8.86%), and RT:K103N (6.64%). The DRMs RT:E138A and RT:K103N confer resistance against non-nucleoside reverse transcriptase inhibitors (NNRTI) rilpivirine and efavirenz, respectively, while RT:K219Q is associated with resistance against nucleoside reverse transcriptase inhibitors (NRTI) zidovudine. Similarly, DRM PI:N88D, associated with resistance against protease inhibitors (PI), such as atazanavir/ritonavir, and tipranavir/ritonavir was observed in two treatment-experienced participants, while DRMs PI:M46L, PI:D30N, PI:N83D, PI:K43T, PI:G73S, PI:L33F were seen in one treatment-experienced individual each (Table 3). No DRM against protease inhibitors was observed in ART-naïve individuals. Analysis also showed that 114 (42%) patients with DRMs belonged to the drug-experienced group, while, 15 (30%) belonged to the ART-naïve group (Table 3).

**TABLE 3:** Classification of the Drug resistance mutations.

**Drug**		**Mutation**	**Naïve, *N* (%) (*n* = 50)**	**Experienced, *N* (%)** **(*n* = 271)**	**Mutation classification**	**Drug associated with resistance**
		K219Q	4 (8.00)	1 (0.37)	Major	AZT
		M184V	0 (0.00)	2 (0.74)	Major	ABC, 3TC
		M184I	0 (0.00)	1 (0.37)	Major	ABC, 3TC
	**NRTIs**					
		L210W	0 (0.00)	1 (0.37)	Major	AZT
		K70R	0 (0.00)	2 (0.74)	Major	ABC, TDF, AZT
		Y115F	0 (0.00)	1 (0.37)	Major	TDF, ABC
		A98G	0 (0.00)	3 (1.11)	Minor	NVP, EFV
**Reverse Transcriptase Inhibitors (RTIs)**						
		K103N	2 (4.00)	19 (7.0)	Major	EFV, NVP
		K101E	0 (0.00)	1 (0.37)	Major/Minor	NVP, EFV
		E138A	4 (8.00)	35 (12.92)	Major/Minor	ETR, RPV
		E138K	1 (2.00)	0 (0)	Major/Minor	EFV, NVP
		E138G	0 (0.00)	1 (0.37)	Major/Minor	EFV, NVP
		V179L	2 (4.00)	10 (3.69)	Major	EFV, NVP
	**NNRTIs**					
		V179F	1 (2.00)	0 (0)	Minor	EFV, NVP
		Y181C	0 (0.00)	1 (0.37)	Major	EFV, NVP
		G190A	0 (0.00)	2 (0.74)	Major/Minor	EFV, NVP
		V106I	1 (2.00)	2 (0.74)	Minor	NVP
		F227C	0 (0.00)	1 (0.37)	Major/Minor	EFV, NVP
		L234I	0 (0.00)	1 (0.37)	Minor	DOR
		H221Y	0 (0.00)	1 (0.37)	Major	EFV, NVP
		M46L	0 (0.00)	1 (0.37)	Minor/Major	LPV/r
		D30N	0 (0.00)	1 (0.37)	Major	NFV
	**Major**					
		N88D	0 (0.00)	2 (0.74)	Minor	ATV/r, SQV/r, NFV
**Protease Inhibitors (PIs)**						
		N83D	0 (0.00)	1 (0.37)	Minor/Major	ATV/r, TPV/r
		G73S	0 (0.00)	1 (0.37)	Minor	LPV/r
	**Minor**					
		L33F	0 (0.00)	1 (0.37)	Minor	LPV/r

*The mutation classification column, indicates mutations that are classified as major or minor DRMs. The last column shows the association of a mutation with resistance to a particular drug, as indicated in the Stanford drug resistance database and/or International AIDS Society (IAS) 2019 report, respectively, while bold drug names indicate the major resistance by IAS 2019. The abbreviations of the antiretroviral drugs are as follows: NRTI; zidovudine (AZT), lamivudine (3TC), abacavir (ABC), tenofovir (TDF), etravirine (ETR), rilpivirine (RPV). NNRTI; efavirenz (EFV), nevirapine (NVP), doravirine (DOR). PI: lopinavir/ritonavir (LPV/r), nelfinavir (NFV), atazanavir/ritonavir (ATV/r), tipranavir/ritonavir (TPV/r), Saquinavir/ritonavir (SQV/r). % = percentage of total participants.*

Next, sequences with any DRMs were analysed for clustering. Among the 78 sequences from the Larkana outbreak with prevalent drug resistance mutations, the DRM RT:K219Q was shared between five sequences whereof all clustered together in cluster_CRF02_AG_1 (Tables 2, 3). The DRM RT:M184V was found in two sequences – both in cluster_CRF02_AG_1. The DRM RT:K103N was found among 21 sequences, whereof 19 sequences were found in cluster (cluster_CRF02_AG_1). The two remaining sequences were not part of any cluster. The DRM RT:E138A was found in 38 sequences of different subtypes including 21 sequences in cluster_A1_1, three sequences in cluster A1_2, seven sequences in cluster_CRF02_AG_1, one sequence in cluster_CRF02AG_2, and six sequences that were not part of a cluster. The DRM RT:V179L was distributed among 10 sequences in cluster_CRF02AG_1, and two sequences in cluster_CRF02AG_2 (Tables 2, 3).

## Discussion

In this study, we determined the HIV-1 subtype distribution, phylodynamics, and presence of HIV-1 drug-resistance mutations of the 2019 HIV-1 outbreak among children in Larkana, Pakistan. Seventeen distinct clusters were found among the Larkana outbreak sequences, indicating that HIV-1 was introduced from multiple sources rather than from a single source, as previously suggested (Arif, 2019; Siddiqui et al., 2020). A similar large-scale nosocomial HIV-1 outbreak was reported in Libya 1998–1999 (Visco-Comandini et al., 2002), where a monophyletic HIV-1 CRF02_AG cluster was identified among children visiting the El-Fatih Children’s hospital in Benghazi. The HIV-1 transmission in the Libyan children was suggested to originate from contaminated intravenous injections (although not from blood or blood products) (Visco-Comandini et al., 2002). Similarly, the HIV-1 transmissions in the 2019 Larkana outbreak were strongly associated with visits to both public and private sector facilities, but not with a single healthcare facility, and with receipt of infusions, injections and blood transfusions (Mir et al., 2020b), implying transmission through poor infection control practices. Some of the children had HIV-1 positive mothers, raising the possibility of mother-to-child HIV-1 transmission. However, the possibility of vertical transmission could not be verified due to unavailability of maternal samples. Taken together, our results indicate that the Larkana outbreak was not the result of a single-source transmission from one health care practitioner, but may have resulted from through multiple sources at different health facilities. Moreover, and as previously suggested, our results indicate that poor infection prevention control is still present in Larkana (Altaf, 2018).

The HIV-1 subtype analysis showed a high prevalence of CRF02_AG and sub-subtype A1. This is consistent with previous reports indicating sub-subtype A1 as the dominant circulating subtype in Pakistan (Khan et al., 2018), whereas CRF02_AG has shown increasing prevalence more recently (Chen et al., 2016). A study reporting on the molecular epidemiology of HIV-1 in Pakistan suggested that sub-subtype A1 was introduced in Pakistan 1989 (95% HPD: 1984–1994) (Chen et al., 2016). After this introduction, sub-subtype A1 disseminated rapidly to become the dominant HIV-1 strain (Chen et al., 2016). However, no information exists about the introduction of CRF02_AG in Pakistan, although it has been shown that the prevalence of HIV-1 CRF02_AG infections are increasing – especially in high-risk populations (Cholette et al., 2020). It is therefore possible that HIV-1 CRF02_AG become the dominant HIV-1 strain in Pakistan over time.

In our analysis, the tMRCA for the two large clusters were both estimated to 2016 (CRF02_AG: 95% HPD interval: 2015–2017; A1: 95% HPD interval: 2015–2018), suggesting ongoing HIV-1 transmissions several years prior to the 2019 outbreak. Interestingly, the estimated tMRCA of the two main clusters coincides with the time of the previously reported HIV-1 outbreak in Larkana in 2016, which also occurred in a nosocomial setting (Brenner et al., 2007; Siddiqui et al., 2020). Furthermore, the majority (80%) of children identified in the outbreak had stage 3 or 4 disease (the moderately and severely symptomatic stage, respectively, mostly associated with chronic to acute-chronic infection) (Weinberg and Kovarik, 2010; Altaf et al., 2016), indicating that some of the HIV-1 infections identified in the 2019 outbreak occurred a few years prior to 2019. Moreover, no reference sequence from the global HIV-1 epidemic was found in the Pakistani clusters, suggesting a localised HIV-1 epidemic. It is possible that the transmission may have been ongoing within healthcare settings, and the active screening programme implemented by the provincial AIDS Control Program in response to the initial diagnosis of HIV-1 in 14 children in 2019 identified these infections.

Phylogenetic analyses demonstrated no mixing between the outbreak sequences and sequences previously obtained from other high-risk groups in Pakistan. This was further supported by dating of the outbreak and other Pakistani clusters, indicating that the splitting time points between the outbreak sequences and that of other high-risk groups in Pakistan occurred between 1994 and 2012 (combined HPD interval). This suggests that HIV-1 most likely were introduced in Larkana between 10 and 27 years ago, and that the HIV-1 transmissions are now localised to certain regions of Larkana. It is possible that HIV-1 arose from nosocomial routes through the widespread poor infection prevention control practices in healthcare settings and contaminated blood (Cotton and Rabie, 2020; Mir et al., 2020a).

DRM analysis showed the presence of multiple DRMs associated with resistance against reverse transcriptase inhibitors, some of which were shared among sequences in the identified clusters. The presence of shared DRMs may indicate transmission of drug-resistant strains in outbreak sequences. The most prevalence DRM identified among both ART-experienced and ART-naïve individuals was RT:E138A which confers a high-level resistance to NNRTIs such as Rilpivirine (Jeulin et al., 2014). A high prevalence of this mutation has been seen previously in ART-experienced PLWH in Pakistan (Shah et al., 2011) and when found in the ART-naïve population, it may be present due to transmission from non-compliant ART-experienced individuals. The presence of DRMs, especially associated with resistance to zidovudine and nevirapine, two out of three that are drugs part of the ART regimen given to the children, may lead to ART failure (correlating with failure to suppress the viral load), and increase the possibility that these individuals may acquire severe form of disease (Gupta-Wright et al., 2020). A second DRM common in the outbreak sequences was the RT:K219N mutation, a thymidine analogue mutation associated with potential low-level resistance against zidovudine (Rhee et al., 2003). Presence of DRMs and spread of strains containing the mutation may lead to first line medications becoming obsolete and present challenges due to limited availability of second-line medications.

The strengths of the study are a relatively good sample size, active collection of samples during the outbreak and a comprehensive phylogenetic and phylodynamic analysis of the Larkana outbreak to identify subtype distribution and evolutionary relationship between sequences. The limitations include amplification of a single gene (*pol*) only and short sequence length. While the parent case-control study showed that visits to both private and public sector health facilities and higher frequency of injections was associated with HIV-1 infection, we were unable to correlate the dyads and clusters with geographical data. The lack of samples of all biological mothers, where the mother was also HIV-positive, precluded investigation of the role of mother-to-child transmission. Future studies based on HIV-1 sequences sampled from different years (and HIV-1 risk groups) could shed light on the effectiveness of ART programs in Pakistan and provide an even more detailed picture of the Larkana outbreak.

In conclusion, our study findings showed that the Larkana paediatric outbreak did not originate from a single source and is likely a consequence of ongoing transmission within the high-risk groups of Larkana and introduced into the exposed individuals at risk of acquiring through poor infection prevention control practices in healthcare settings. Furthermore, the presence of multiple drug resistance mutations in the strains circulating in Larkana, especially to first-line ART drugs, is worrying as it limits treatment options. Large-scale transmission of resistant strains can hamper Pakistan’s efforts to achieve the 90-90-90 goal (Maddali et al., 2016). These findings highlight not only the urgent need to improve blood safety and infection prevention control, but also the need for comprehensive molecular epidemiological studies and molecular surveillance to understand the distribution of different genotypes as well as origin, transmission, and drug resistance patterns.

## Data Availability Statement

The datasets presented in this study can be found in online repositories. The names of the repository/repositories and accession number(s) can be found below: https://www.ncbi.nlm.nih.gov/genbank/, MN698251, MN698252, MN698253, MN698255, , MN698257, MN698258, MN698259, MN698260, MN698261, MN698262, MN698263, MN698264, MN752136, MN752137, and MT748850–MT749178.

## Ethics Statement

The studies involving human participants were reviewed and approved by Aga Khan University Ethics Review Committee (ERC #2019-1536-4200). Written informed consent to participate in this study was provided by the participants’ legal guardian/next of kin.

## Author Contributions

SA, RF, and FM conceived the study. SA, GN, DS, and WR performed the experiments. SA and GN wrote the first draft. SFM, AS, ShS, SM, SaS, SA, RF, PK, JE, and FM were involved in outbreak investigation. GN, DS, and AN cleaned the data and prepared the final datasheets. SA, GN, and DS have equal contributions in all experiments and data analysis. JE, RF, and FM had an equal contribution in project supervision. All authors contributed to the article and approved the submitted version.

## Conflict of Interest

The authors declare that the research was conducted in the absence of any commercial or financial relationships that could be construed as a potential conflict of interest.

## Publisher’s Note

All claims expressed in this article are solely those of the authors and do not necessarily represent those of their affiliated organizations, or those of the publisher, the editors and the reviewers. Any product that may be evaluated in this article, or claim that may be made by its manufacturer, is not guaranteed or endorsed by the publisher.

## Footnotes

1
http://www.hiv.lanl.gov

2
https://github.com/rambaut/figtree/releases
